# Medical and Philosophical Intelligence

**Published:** 1818-01

**Authors:** 


### MEDICAL AND PHILOSOPHICAL INTELLIGENCE.

THE following letter arrived after the first proof of our Intelligence had been struck off, and, in conformity with the wish of the learned writer, we made a fresh arrangement of our matter, to give as much and as early publicity as possible to a communication from such a source.

*To the Editor of the London Medical and Physical Journal, No. 73, St. Paul's Church-yard: (to be forwarded without delay.)*

SIR,—The tenor of your note, subjoined to my communication in your last Number, may render it doubtful, with many of your readers, whether it is not incumbent upon *me* to commence the investigation of the late Mr. J. Hunter's claim to the doctrine of "*pus being a secretion.*" You say—"We shall be extremely thankful to Dr. Curry, by the honour of a further communication on this interesting subject; and we trust Mr. Hunter's friends will not be backward in defending their master.—EDIT."

Now, Sir, although I shall be the last to shrink from any engagement, whether expressed or implied, that I have once entered into, it is certainly not MY *part* to commence the attack on this occasion; but, on the contrary, it is clearly incumbent on Mr. Hunter's friends to support *his* claim to the *honour* (whatever it may be), by proving that he taught in his *Lectures*, or promulgated in some other way, the theory in question, previous to the year 1763, when Dr. Morgan published his Inaugural Dissertation at Edinburgh, entitled *de Puris Confectione*, in which he has stated the arguments and conclusions for this theory in as formal and complete a manner as could be required to give him the claim to priority. When Mr. Hunter's friends have substantiated *his anterior right*, *then*, and *not till then*, will it be at all necessary for *me* to enter upon the more important question of its *truth* or *falsity*.

I am, Sir, your very obedient servant,

JAMES CURRY.

*Bridge-street, Blackfriars; Sept.* 25, 1817.

*** The only notice we can at present take of a communication with which we are so recently honoured, is, to request that Dr. Curry will favour us with a copy of the passage in Dr. Morgan's Dissertation, and, if not too much trouble, with his translation of it. The first we request, because an Inaugural Dissertation, especially more than fifty years old, is in few hands; the latter, inasmuch as there may be some obscurity in the Latin, or those disciples of Mr. Hunter, who are best acquainted with his opinions, may, like their master, be the least acquainted with the dead languages.

*To the Editors of the London Medical and Physical Journal.*

Gentlemen,—You will pardon the liberty I am about to take with the pages of your admirable Journal, in requesting a portion for the information of one ignorant of any source equally authentic. —I am the father of a large family in the north of England, and have one son, whose inclination bends towards the medical profession. The branch chosen is that of a physician; he is now nineteen years of age, and has been three years with a surgeon of extensive practice, chiefly among the lower class of people, affording him greater opportunities of practical improvement than he could have gained among the more opulent.

In consequence of the death of his master, which happened a little before the expiration of his apprenticeship, he is now in London, walking the hospitals; and, at the end of two years, I purpose sending him to Edinburgh. Permit me to say, that he has acquired a tolerably good classical education, having had a tutor during his apprenticeship. Now, if any of your correspondents will inform me how long it will be necessary for him to remain in Edinburgh, after he shall have completed his studies in London; what knowledge of the classics is required; what are the books used in the examination; some particulars relative to the Thesis Inauguralis, and any others which their knowledge of the subject will suggest, they will infinitely oblige,

*Dec.* 15, 1817. Pater-Familias.

*** We have received, at different times, letters from young gentlemen, and we always keep them in view in our general reference to Juvenis. But this is the first application from a source, in our opinion, requiring infinitely more attention, inasmuch as we are now to address those who can have no knowledge of the subject but what they receive from us. We, therefore, withhold any reply till we have taken time for due consideration, and shall be thankful for the assistance of the *alumni* of any of the universities in the United Kingdoms, as well as of the general practitioner.

---

*To the Editors of the London Medical and Physical Journal.*

Gentlemen,—Having heard that considerable efficacy has been ascribed to the use of sweet-oil, taken in the proportion of half an ounce a day for six weeks previous to child-birth, and conceiving that you will be decidedly of opinion, that, whatever may tend to alleviate the female sufferings on such occasions should be made generally known, I beg to be informed, through your valuable Journal, if it is reasonable to attribute any good effects to such regimen. I trust, Gentlemen, that the importance of this subject will apologise for my calling your attention to it; and I am the more earnest in requesting your opinion, from having been credibly informed that there are many instances of ladies, who before had experienced most painful and dangerous labours, having, from this simple administration, been delivered with comparative ease.

I have the honour to be, Gentlemen,

Your obedient servant,

Q. R.

The few words inserted in our last concerning Mr. Want's medicine, impressed us with an idea that Mr. Want had discovered some fallacy in his own remedy. The following is an extract of a letter from Mr. Wilson :—

"Mr. Want's assertion, that my medicine is prepared according to his prescription for making the French medicine, is untrue."

We here give notice that the subject cannot be continued in the London Med. and Phys. Journal.

---

Dr. Magendie lately read to the Royal Academy of Sciences a very interesting paper on the effects of Prussic acid, both as a poison and as a medicine. Laurel-water, which owes its properties to this acid, has been long known in both these respects. Some experiments had been performed on Prussic acid, as prepared by Scheele, which prove it to have similar, but more powerful, effects. Mr. Guy Lassac's mode of preparation renders this acid much stronger: it is about sixty times more concentrated than Scheele's acid; but, however one may be disposed to expect a great difference in their effects from their different degree of concentration, its energy surpasses all that could be imagined;—it is, beyond comparison, the most dreadful poison known. When properly diluted, it also acts as one of the most energetic and useful medicines. Dr. Magendie has employed it with great success in many cases of affections of the breast, known by the name of nervous coughs; but its utility is far from being confined to similar complaints. From a certain number of cases, inadequate to decide the question, but sufficient to give the liveliest hopes, it is likely to have the greatest effect in phthisis, not only as a most powerful palliative, but even as capable of curing this destructive disease in an early stage.

---

### *Effect of Lightning on a Tree.*

*George-street, Edinburgh; July* 23, 1817.

In my walk two days ago, I happened accidentally to step into Craiglockart Garden, near the village of Slateford, about two miles west of Edinburgh, where, after inspecting the garden, hot-houses, and romantic grounds, upon the wooded banks of the water of Leith, the keeper informed me that the thunder-storm of the 10th ult. had been particularly terrible at that spot, that the lightning had struck a tree on the side of the highway to the north of the garden wall, and had afterwards struck a man who had taken shelter in a neighbouring out-house attached to the back of the garden, near the tree.

Wishing to ascertain the appearance of a tree struck with electric fluid (having often observed large blotches on the bark of trees said to have been caused by the lightning), I requested the keeper (Mr. Robertson) to show me the tree. I found it to be an English elm. The lightning had struck it at a small decayed knob about 15 feet above the ground, on the north side of the tree. It descended all round in a spiral form, and went off from the ground, destroying a large quantity of nettles and grass at the root of the tree in a south-west direction, towards the house where the man had taken shelter, where at the root of the tree I observed it had torn up the earth, which is still quite apparent, from the root of the tree, in the shape nearly of a large grooved wheel track, diminishing in size as it extends outward. From this it entered the out-house where the men were, and struck one of them so much that he was quite stupified for some time; and afterwards, as the men relate, the lightning went off by a skylight, although there is no visible mark of its course after quitting the root of the tree, and the grooved way, which does not extend above two feet from the tree, but is in the direction of the door of the house. In this house there was a considerable quantity of iron. Thus it would appear that leading a conductor to the earth is no certain rule of safety—the lightning does not descend into the ground.

The appearance of the effects of the fire upon the tree is quite different from the blotches which, in my ignorance, had frequently been passed upon me for the effects of lightning, and which probably is some disease of the tree, or animalculæ. The appearance of this tree is as if the top knobs of the outer cortex had been touched with a wright's plain, having a white glistening colour, as if after friction. No black traces remained upon the tree, and nothing bore the resemblance of burning, except some lateral branches, and the nettles and brushwood at the bottom of the tree.

The most surprising fact, however, is, that four years ago two trees (within a few yards of the elm that was struck) were in like manner successively struck with lightning. The one tree was a beech, and is now cut down, having decayed by the effects of the lightning; the other a fir, I believe, which remains a wretched spectacle to this day. This led me to inquire as to the contiguous metals imbedded in the ground under the spot.

Fortunately a well, about 18 feet deep, is sunk close by, which a man informed me he had been at the bottom of, and he assured me that the only mineral cut through in that depth was a greyish free-stone.

I have thought it proper to set down this information for you as I received it, and saw it: and, if it can throw any light, or afford any hints to your learned friends, upon this most awful phenomenon of nature, I shall be happy. The shape of the groove by which the electric fluid escaped from the tree may, perhaps, be some foundation for ascertaining the form of the forked lightning. And from the direction as marked on the ground, and the circumstance of the man being so sensibly affected in the out-house, it would appear to have been the same flash that did both. JOHN GOVAN.

*(From Dr. Thomson's Annals of Philosophy.)*

The following Tables show the state of health in the second city of importance in Ireland.—

Return of Patients admitted into the Fever-House, within the last Fifteen Years.

| Years. | Admitted. | Discharged. | Died. |
|---|---|---|---|
| 1803 | 254 | 241 | 9 |
| 1804 | 190 | 185 | 4 |
| 1805 | 200 | 192 | 7 |
| 1806 | 441 | 427 | 13 |
| 1807 | 192 | 188 | 4 |
| 1808 | 232 | 225 | 7 |
| 1809 | 278 | 267 | 6 |
| 1810 | 432 | 403 | 17 |
| 1811 | 646 | 627 | 19 |
| 1812 | 617 | 610 | 13 |
| 1813 | 550 | 521 | 26 |
| 1814 | 845 | 814 | 27 |
| 1815 | 717 | 705 | 21 |
| 1816 | 1026 | 958 | 37 |
| 1817 | 2707 | 2503 | 100 |
| Total | 9327 | 8866 | 310 |

The following Return shows that the fever had progressively increased up to the month of November last.

Return of Patients admitted into the House of Recovery, from the 8th November 1816, to the 8th November 1817, both days inclusive.

| Years. | Dec. | Jan. | Feb. | Ma. | Apr. | May | June | July | Aug. | Sep. | Oct. | Nov | Total |
|---|---|---|---|---|---|---|---|---|---|---|---|---|---|
| 1816-17 | 124 | 144 | 146 | 160 | 170 | 204 | 219 | 224 | 262 | 265 | 414 | 425 | 2707 |

| | |
|---|---|
| In the House on the 8th November, 1816 | 47 |
| Admitted from 8th November 1816, to 8th November 1817 | 2707 |
| | 2754 |
| Discharged, cured | 2503 |
| Died | 100 |
| Remain in the house | 151 |
| | 2754 |

The number of deaths, in the aggregate of the first table, is about 1 in 30; in the last, about 1 in 25. In a subsequent table, it appears, that the house, though built for the reception of only 121 patients, contains at this time 148. This is a most dangerous addition, and it seems difficult to conceive why some of the large empty houses, which we are told are numerous in Cork, are not occupied in aid of the hospital, which, if once so crowded as to generate an infectious atmosphere, will be worse than useless.

---

How much we regret that the nature of our work will not permit room for the whole of what, in the Journals devoted to Philosophy in general, is, with much justice, called the "Triumph of Science."

"The subjoined decided and honourable testimony given to the originality and utility of Sir Humphry Davy's discovery of the Safety-Lamp for Miners, deserves to have a more durable record than the ephemeral columns of a newspaper, and our readers, we are sure, will therefore thank us for giving it a place in our pages.

"The coal-owners of the rivers Tyne and Wear, the body most extensively benefited by Sir Humphry Davy's safety-lamps for preventing explosions in coal-mines, have shown their sense of the importance of the discovery to their interests, and those of humanity, by presenting Sir Humphry with a very handsome service of plate, of the value of nearly two thousand pounds. The ceremony of the presentation of it took place on Saturday, October the 11th, when a grand dinner was given to Sir Humphry by the coal proprietors and owners, at the Queen's Head at Newcastle, where the plate was exposed for public inspection, and the designs, taste, and execution, equally admired. J. G. Lambton, esq. M.P. for the county of Durham, was in the chair. There were present—the mayor, sheriff, and town-clerk, of Newcastle; the Rev. Dr. Gray, J. Collinson, and J. Hodgson ; Messrs. Warren, Lamb, Baker, Lorraine, Buddle, Ellison, Botts, Brown, Mowbray, Robinson, and about fifty other gentlemen."

---

The last number of Annals of Philosophy contains the following paragraph:—

"Miss M'Avoy is still the great subject of discussion at all the parties in Liverpool : the chief difficulty seems to be to *blind* her completely; the goggles (goldbeaters'-skin, with a black patch over it,) not being satisfactory. ———— tried them on, and found that, from their not fitting quite tight to the eye, she could see, and did tell colours, and read, as Miss M'Avoy does."

We know not whether the person alluded to by ————, be Mr. Martin, and the female pronoun an error of the printer ; as we have heard that a gentleman of that name could use the above apparatus in such a manner as not to prevent his seeing.—Is this history now matured into the somewhat incomprehensible statement in the above extract?

We have also heard that, when Miss M'Avoy, putting her hands behind her, is able to tell the hour by the watch placed before her fingers, there are looking-glasses in the room, which reflect the watch to her eyes. We confess we should more readily believe that she saw with her toe-nails, than that the gentlemen who have witnessed her powers could be so duped.

We much admire the candour of Dr. Brandreth, if the account we hear is true, that he acknowledges himself convinced whilst in her presence, but cannot help doubting whilst absent. In our next, we shall give an account of Dr. Renwick's publication, and several other ophthalmic articles, without offering any opinion of our own.

---

In our Retrospect, we have been somewhat hard on the continental Societies for overlooking our illustrious countryman. It is with much satisfaction we have just read a letter from Paris, in which we are informed that, at a late meeting of the Institute, M. Petit (we believe at the suggestion of Cuvier) read a long eulogium on John Hunter, in which he gave every praise to the originality of his genius,—the extent and accuracy of his experiments,—the clearness of his deductions,—the important results deduced from them in every branch of physiology, particularly pathology. What may we not expect from French industry, when corrected by that constant attention to the laws of animal life, of which Mr. Hunter never suffered himself, in a single instance, to lose sight?

---

We omitted to notice, in our Retrospect, an important article, expecting to find room for it in the Analytical Department. We refer to Mr. Baillie's interesting publication on the virtues of Belladonna in Tic Doloureux, and other cases of Neuralgia, to use the language of the writer. The article is ready for the press, and shall appear in our next.—The same want of room must apologise for deferring a part of the Medico-Chirurgical Transactions.

---

[*Adv.*]—Dr. Adams will commence his Spring Course of Lectures on the Institutes and Practice of Medicine, at his house in Hatton-garden, the first Tuesday in February, at ten o'clock in the morning precisely. The students, during their attendance on the course, will have the privilege of seeing the practice of the Small-pox Hospital, and of the New Finsbury Dispensary.

---

[*Adv.*]—Middlesex Hospital.—Dr. P. M. Latham and Dr. Southey will begin their next Course of Lectures on the Practice of Physic, at this hospital, on Wednesday, the 21st of January, at nine in the morning.

---

Lectures on the Structure and Diseases of the Teeth, &c. will commence on Friday, the 9th of January, at half after five o'clock.

A Course of Lectures on the Anatomy, Physiology, and Pathology of the Ear, will commence on Thursday, January 15th, at seven o'clock in the evening.

A Course of Lectures on Anatomy, Physiology, Pathology, and Surgery, will commence on Saturday, January 24th, at eight o'clock in the evening precisely, and be continued every Tuesday, Thursday, and Saturday, at the same hour.

A Course of Lectures on Surgery will commence on Monday the 19th January, at eight in the evening; to be continued on Mondays, Wednesdays, and Fridays.

An Introductory Lecture to Dr. Uwins' Spring Course on the Theory and Practice of Medicine, will be delivered on Friday, the 30th of January, at seven o'clock in the evening precisely. The lectures will be continued, at the same hour, every Monday, Wednesday, and Friday, until the conclusion of the course, which will be about the end of April.

### REPORT OF DISEASES.

The mild season has been extremely favourable to the public health. Winter coughs and asthmas have scarcely appeared, and always in a mild form. Rheumatism, the continual bane of poverty, has had a very confined influence; and the chronic diseases, in general, have scarcely suffered the customary exacerbation from the return of the season.

The fevers are still numerous, though for the most part mild. But this is only in the recesses of poverty, the inhabitants of which are gradually inured to an atmosphere of their own creating, and are prepared, by previous privations, for escaping the dangers of that increased action which the exposure to the infectious atmosphere induces in subjects better fed and in higher health. A remarkable instance of this kind has occurred in the north-west part of the town. A female, from one of the houses of poverty, with her clothes contaminated by this infection, paid a visit to a family by whom she was patronised. The kind disposition of the females induced them to give their *protegée* that sort of reception which softens the miseries of poverty, and does so much honour to the heart of unsuspecting youth. The consequence was, that, at an unusually short period afterwards, all of them, four in number, were seized with symptoms of fever, attended with delirium. The eldest, being at an age when all the actions of life are the most powerful, had the symptoms most severely, and died early in the disease. None of the family, excepting these four, were affected, though the latter were, of course, carefully nursed.

Among the poor, in whom febrile symptoms are milder, sore throats and sore mouths are very frequent. They seem to arise from the same tainted atmosphere, producing a local effect.

The bills of mortality for the year return the same number of deaths by fever as on the preceding year; but these disgraceful archives of the first metropolis in the world afford no criterion of its health. We before remarked, the fever among the poor, who have felt it more generally, has been comparatively mild. Besides which, scarlet fever is included in the general list of fevers of all descriptions. Not only are Mary-la-bonne and Pancras excluded from the Bills, but, in the most populous parishes, the numbers buried at dissenting cemeteries often exceed those in the consecrated grounds. In our next, we shall offer some further remarks on the mortality of the year.

### A METEOROLOGICAL JOURNAL,

By Messrs. William Harris and Co. 50, Holborn, London.

*From the 20th November to the 19th December, inclusive.*

| Day of Month. | Moon. | Rain gauge | Therm | | | Barom. | | De Luc's Hygrom. Dry | Damp | | Wind. | | Atmospheric Variation. | | |
|---|---|---|---|---|---|---|---|---|---|---|---|---|---|---|---|
| Nov. | | | | | | | | | | | | | | | |
| 20 | | | 43 | 45 | 40 | 30 | $29^{9}$ | | 4 | 4 | W | W | Fine | | |
| 21 | | | 42 | 47 | 42 | $29^{8}$ | 30 | | 2 | 2 | W | NW | Fine | | |
| 22 | | | 42 | 44 | 43 | 30 | $30^{1}$ | | 1 | 1 | N | SW | Fine | | |
| 23 | ○ | | 43 | 45 | 45 | $30^{1}$ | $29^{9}$ | | 2 | 2 | W | SW | Clo. | | Fine |
| 24 | | ,07 | 45 | 45 | 36 | $29^{8}$ | $29^{7}$ | | 2 | 1 | SW | W | Clo. | | |
| 25 | | | 37 | 39 | 37 | $29^{9}$ | $30^{1}$ | | 1 | 1 | W | SW | Fine | | Rain |
| 26 | | ,18 | 40 | 45 | 44 | $30^{1}$ | $30^{1}$ | | 1 | 4 | SW | SW | Rain | Clo. | Fine |
| 27 | | ,12 | 47 | 51 | 44 | $30^{1}$ | $30^{1}$ | | 4 | 1 | SW | SW | Clo. | Fine | |
| 28 | | | 46 | 48 | 45 | $30^{1}$ | $29^{9}$ | | 2 | 2 | SW | W | Fine | Clo. | Fine |
| 29 | | | 46 | 51 | 50 | $29^{9}$ | $29^{9}$ | | 2 | 3 | SW | SW | Clo. | | Rain |
| 30 | | ,13 | 52 | 55 | 52 | $29^{9}$ | $29^{9}$ | | 3 | 5 | SW | SW | Clo. | | Rain |
| Dec. | | | | | | | | | | | | | | | |
| 1 | ◑ | | 53 | 54 | 47 | $29^{8}$ | $29^{7}$ | | 6 | 7 | SW | NW | Rain | | |
| 2 | | | 47 | 51 | 35 | $29^{6}$ | $29^{5}$ | | 7 | 6 | SW | NW | Rain | Clo. | Fine |
| 3 | | | 36 | 40 | 33 | $29^{5}$ | $29^{7}$ | | 0 | 0 | WNW | W | Rain | | |
| 4 | | | 34 | 38 | 37 | $29^{9}$ | $29^{8}$ | | 0 | 0 | NNE | S | Clo. | | |
| 5 | | | 40 | 42 | 40 | $29^{7}$ | $29^{5}$ | | 1 | 4 | S | SSW | Clo. | | |
| 6 | | | 41 | 43 | 35 | $29^{4}$ | $29^{4}$ | | 5 | 3 | SW | SW | Clo. | Fine | |
| 7 | | | 37 | 41 | 39 | $29^{4}$ | 28 | | 2 | 2 | SW | W | Fine | Clo. | Rain |
| 8 | ● | | 41 | 42 | 38 | $28^{7}$ | $28^{8}$ | | 2 | 2 | W | NW | Rain | | |
| 9 | | | 39 | 43 | 32 | 29 | $29^{1}$ | | 2 | 3 | NW | N | Rain | Fine | |
| 10 | | | 34 | 39 | 29 | $29^{3}$ | $29^{5}$ | | 1 | 0 | W | W | Snow | Fine | |
| 11 | | | 32 | 34 | 27 | $29^{6}$ | $29^{6}$ | | 0 | 0 | W | SW | Fine | | |
| 12 | | | 27 | 30 | 35 | $29^{7}$ | $29^{6}$ | | 0 | 0 | W | SE | Fog. | | Rain |
| 13 | | | 37 | 41 | 38 | $29^{6}$ | $29^{6}$ | | 2 | 5 | SE | E | Rain | | |
| 14 | | | 40 | 45 | 35 | $29^{5}$ | $29^{5}$ | | 5 | 8 | SE | SW | Rain | Clo. | |
| 15 | ◐ | | 37 | 41 | 40 | $29^{7}$ | $29^{7}$ | | 7 | 6 | SE | SSW | Fine | | Rain |
| 16 | | | 41 | 47 | 39 | $29^{7}$ | $29^{6}$ | | 5 | 7 | S | S | Rain | Clo. | Rain |
| 17 | | | 40 | 42 | 40 | $29^{6}$ | $29^{6}$ | | 10 | 10 | SW | SW | Rain | Clo. | Rain |
| 18 | | | 41 | 44 | 38 | 29 | 29 | | 13 | 17 | SW | SW | Fine | Rain | Clo. |
| 19 | | | 40 | 45 | 39 | $28^{9}$ | 29 | | 18 | 15 | W | WNW | Fine | | Rain |

The quantity of rain fallen in the month of November is 1 inch and 43-100ths.

